# Supplementary material for: Dual PI3K/mTOR Inhibitor NVP-BEZ235 Leads to a Synergistic Enhancement of Cisplatin and Radiation in Both HPV-Negative and -Positive HNSCC Cell Lines
Source: Cancers (Basel). 2022 Jun 28;14(13):3160. doi: 10.3390/cancers14133160 (PMC9265133; doi:10.3390/cancers14133160)
Supplement: Supplementary file 1 [file cancers-14-03160-s001.zip › cancers-1792114-supplementary.pdf]

Supplementary Materials

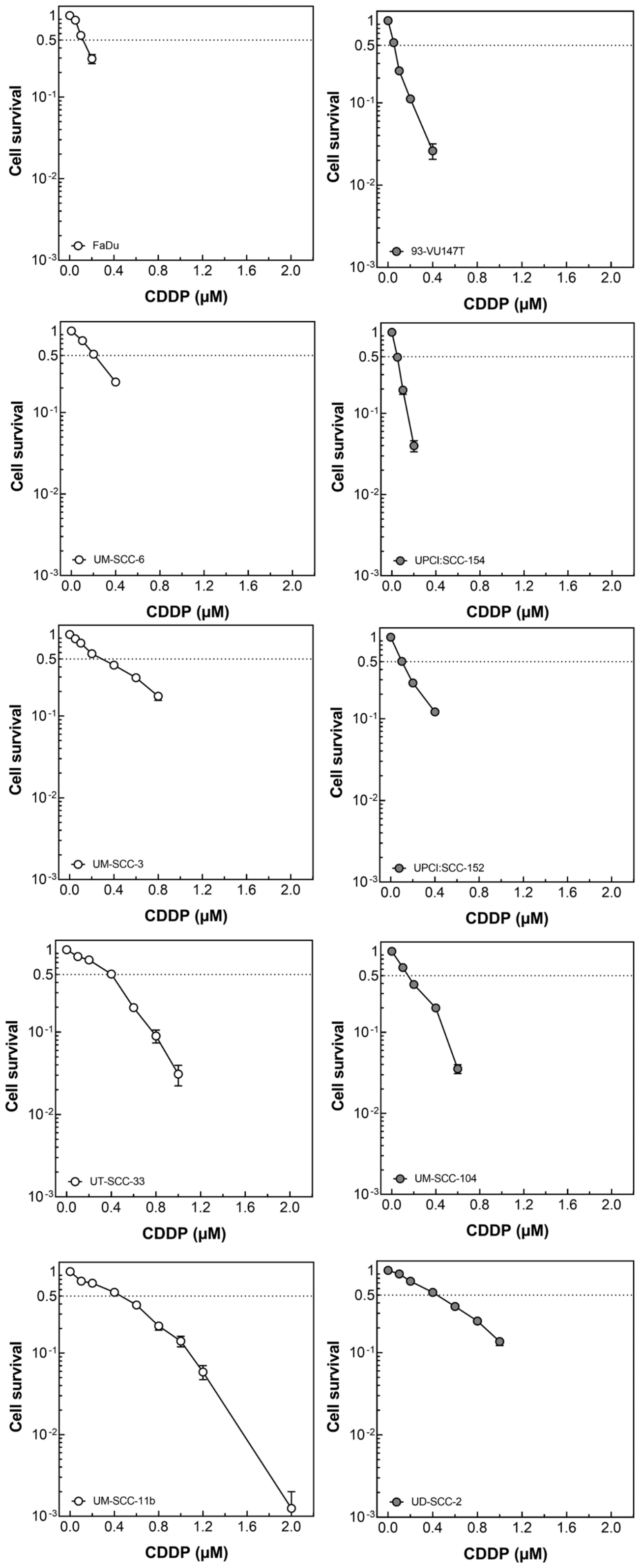

Figure S1. Cell survival after cisplatin (CDDP) of HPV neg. (left) and HPV pos. (right) HNSCC cell lines. Data are presented as mean values ± SEM, n≥3. Dotted line at 50%.

A

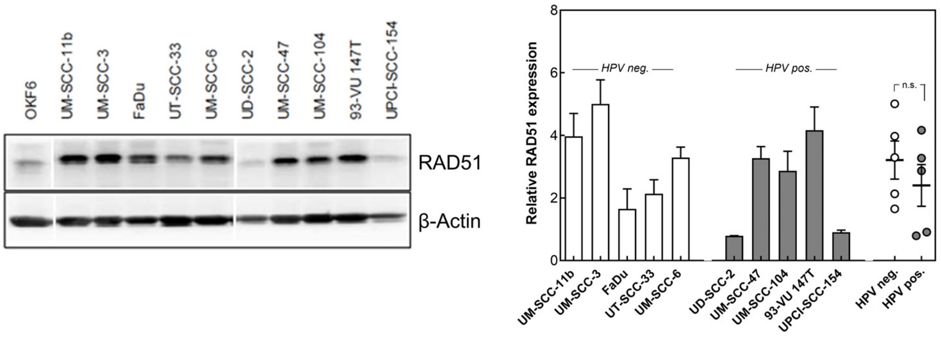

B

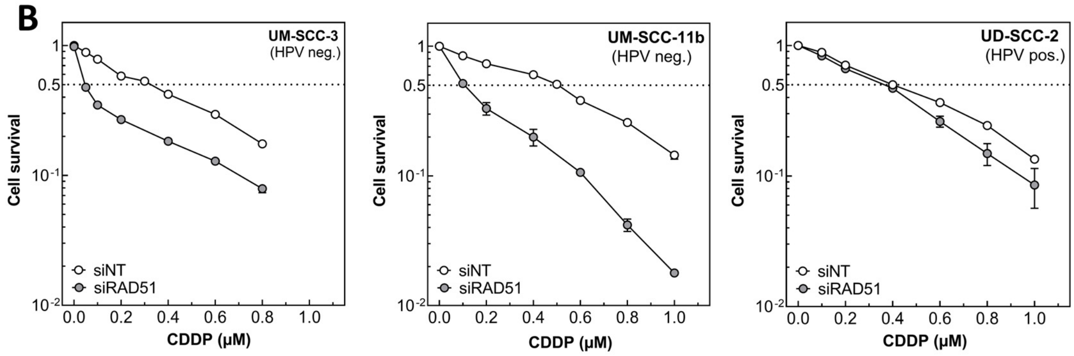

C

Cell line	siRNA	MW of RAD51	MW of RAD51 relative to OKF6	SEM
UM-SCC-3	native	1,100	5,110	0,0040
UM-SCC-3	siNT	1,000	5,010	0,0000
UM-SCC-3	siRAD51	0,137	0,687	0,0265
UM-SCC-11b	native	1,020	3,998	0,0030
UM-SCC-11b	siNT	1,000	3,973	0,0000
UM-SCC-11b	siRAD51	0,118	0,469	0,0022
Cal-33	native	1,056	2,166	0,0070
Cal-33	siNT	1,000	2,110	0,0000
Cal-33	siRAD51	0,130	0,274	0,0061
UM-SCC-47	native	0,985	3,225	0,0188
UM-SCC-47	siNT	1,000	3,273	0,0000
UM-SCC-47	siRAD51	0,177	0,578	0,0465
UD-SCC-2	native	1,399	1,105	0,3799
UD-SCC-2	siNT	1,000	0,790	0,0000
UD-SCC-2	siRAD51	0,099	0,078	0,0515

Figure S2. RAD51 protein expression in HNSCC cell lines and cell survival after RAD51 knockdown and cisplatin. A) Left chart: RAD51 protein expression of HPV neg. and pos. HNSCC cell lines; ß-Actin was used as loading control. Right chart: Mean values ± SEM of relative RAD51 expression in HNSCC cell lines showing that there is no significant difference between HPV neg. and pos. HNSCC cell lines. B) Cisplatin (CDDP) sensitivity of UM-SCC-3, UM-SCC-11b and UD-SCC-2 after knockdown of RAD51 (siRAD51). C) RAD51 protein expression as mean values after densitometric analyses of all western blots after knockdown of RAD51 using siRNA (siRAD51) versus non-target control (siNT) or native cell and the calculated expression of RAD51 relative to OKF6. All data are presented as mean values ± SEM, n≥3.

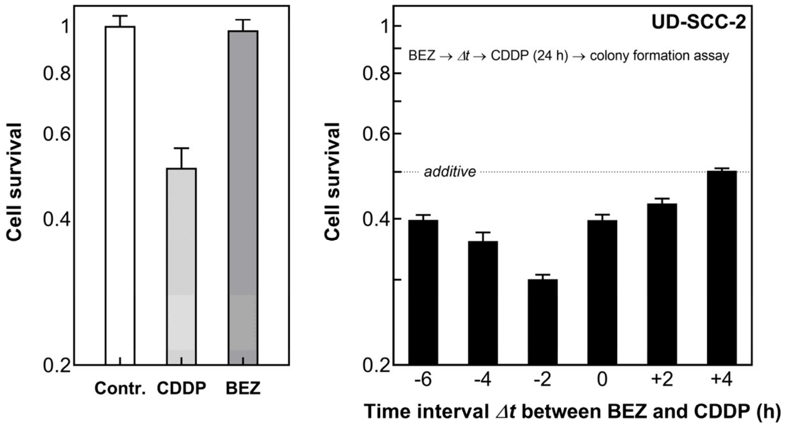

Figure S3. Effect of BEZ235 on UD-SCC-2 cells given at various time intervals (Δt) before or after starting with cisplatin (CDDP) incubation. Left chart: Cell survival of UD-SCC-2 after incubation with cisplatin (IC50) for 24 h or BEZ235 (50 nM) for 26 hours. Right chart: Cell survival after the combined treatment with BEZ235 (50 nM) and cisplatin (IC50) using different time intervals. The strongest effect of the combined treatment was seen when BEZ235 was given 2 hours prior to cisplatin. The dotted line marks the additive effect of BEZ235 and cisplatin. Data are presented as mean values ± SEM, n≥3.

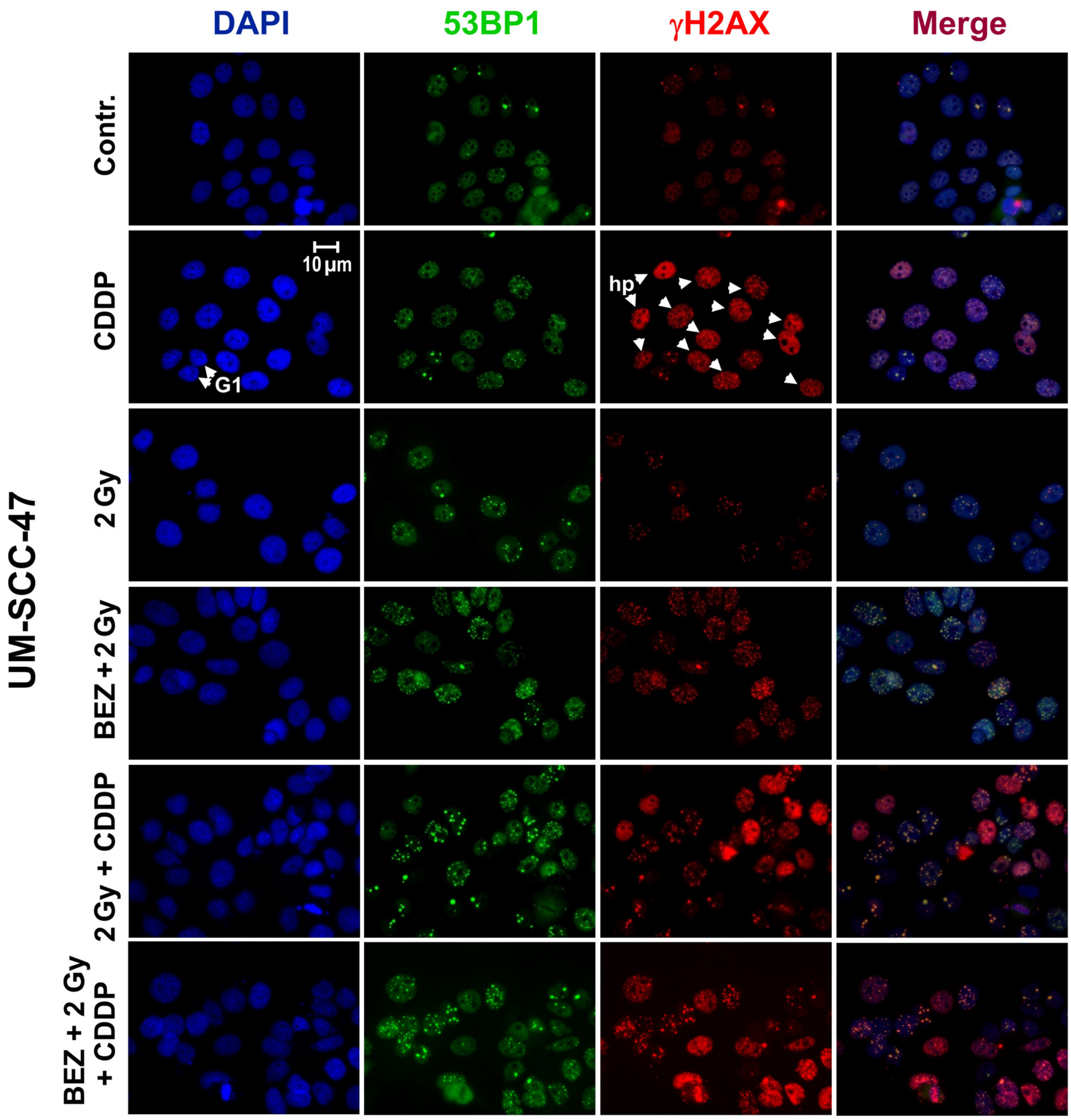

Figure S4. Effect of BEZ235 and the combined treatment with cisplatin (CDDP) and irradiation on γH2AX/53BP1 foci in UM-SCC-47 cells. Cells were treated with/without 50 nM BEZ235 (BEZ) 2 h before irradiation with 2 Gy and cisplatin (CDDP, IC50) treatment. Images as taken for UM-SCC-47 cells; G1 cells, identified by the small nuclear size diameter less than 10 µm; hp, hyper-phosphorylation of γH2AX with > 30 foci.
